# Antibody responses to two new *Lactococcus lactis*-produced recombinant Pfs48/45 and Pfs230 proteins increase with age in malaria patients living in the Central Region of Ghana

**DOI:** 10.1186/s12936-017-1955-0

**Published:** 2017-08-01

**Authors:** Festus K. Acquah, Evans K. Obboh, Kwame Asare, Johnson N. Boampong, Samuel Victor Nuvor, Susheel K. Singh, Michael Theisen, Kim C. Williamson, Linda Eva Amoah

**Affiliations:** 1 Noguchi Memorial Institute for Medical Research, University of Ghana, Accra, Ghana; 2 School of Medical Sciences, University of Cape Coast, Cape Coast, Ghana; 3 Department of Biomedical Sciences, University of Cape Coast, Cape Coast, Ghana; 4 Department of Microbiology and Immunology, School of Medical Sciences, University of Cape Coast, Cape Coast, Ghana; 5 Department for Congenital Disorders, Statens Serum Institut, Copenhagen, Denmark; 6 Centre for Medical Parasitology at Department of International Health, Immunology and Microbiology, University of Copenhagen, Copenhagen, Denmark; 7 Loyola University Chicago, Chicago, IL USA; 8 Uniform Services University of the Health Sciences, Bethesda, MD USA

**Keywords:** Malaria, Transmission-blocking, *Pfs*48/45, *Pfs*230, *Lactococcus lactis*, Seropositive

## Abstract

**Background:**

Recent advances in malaria control efforts have led to an increased number of national malaria control programmes implementing pre-elimination measures and demonstrated the need to develop new tools to track and control malaria transmission. Key to understanding transmission is monitoring the prevalence and immune response against the sexual stages of the parasite, known as gametocytes, which are responsible for transmission. Sexual-stage specific antigens, *Pfs*230 and *Pfs*48/45, have been identified and shown to be targets for transmission blocking antibodies, but they have been difficult to produce recombinantly in the absence of a fusion partner.

**Methods:**

Regions of *Pfs*48/45 and *Pfs*230 known to contain transmission blocking epitopes, 6C and C0, respectively, were produced in a *Lactococcus lactis* expression system and used in enzyme linked immunosorbent assays to determine the seroreactivity of 95 malaria patients living in the Central Region of Ghana.

**Results:**

Pfs48/45.6C and Pfs230.C0 were successfully produced in *L. lactis* in the absence of a fusion partner using a simplified purification scheme. Seroprevalence for *L. lactis*-produced *Pfs*48/45.6C and *Pfs*230.C0 in the study population was 74.7 and 72.8%, respectively.

**Conclusions:**

A significant age-dependent increase in antibody titers was observed, which suggests a vaccine targeting these antigens could be boosted during a natural infection in the field.

**Electronic supplementary material:**

The online version of this article (doi:10.1186/s12936-017-1955-0) contains supplementary material, which is available to authorized users.

## Background

Malaria is still a disease of immense public health importance due to its prevalence and high rates of mortality in the tropics and developing countries, especially in children under five [1]. Global efforts to eliminate the disease have led to an increase in the search for transmission reducing interventions that target the parasite stage required for transmission, called gametocytes, as well as the mosquito vectors. Currently, the main focus of elimination and pre-elimination control measures is to decrease the mosquito vector population as well as control parasitaemia by prompt treatment with effective anti-malarial. Unfortunately, most commonly used anti-malarials, including artemisinin-based combination therapy (ACT), do not effectively eliminate gametocytes and in endemic countries there are also many individuals with asymptomatic infections that never seek treatment and serve as an infectious reservoir. Control efforts would be greatly enhanced by the development of effective vaccines, but to date these efforts have been complicated by antigenic diversity both within and between the distinct stages of the parasite that develop in the human host.

One current strategy is to develop a multi-component vaccine that targets antigens found at different parasite stages, including the stages required for transmission [1]. *Pfs*48/45 and *Pfs*230 are sexual stage antigens that are expressed on the surface of the intraerythrocytic, *Plasmodium falciparum* gametocyte and become exposed on the surface of extracellular gametes in the mosquito midgut [2]. Within 10 min after a gametocyte-infected erythrocyte is taken up in a blood meal by a mosquito, the parasite emerges as an extracellular gamete. Emergence exposes surface-antigens, such as *Pfs*48/45 and *Pfs*230, to antibodies also present in the human blood meal. Both antigens elicit an immune response during a natural infection [3] and *Pfs*48/45 or *Pfs*230-specific antibodies have been shown to block transmission to mosquitoes in a standard membrane feeding assay (SMFA) [4–7]. However, despite their potentially important role in malaria transmission, these antigens have not been included in many field studies due the lack of properly-folded recombinant proteins. Therefore, relatively little is known about the epidemiology of the host response against these antigens. Prior studies have used two-site or competition ELISAs to capture antigen from gametocyte extract using mAb then assaying for the ability of antibodies in human serum or plasma samples to bind or to compete with the binding of a second antibody, respectively [8–10]. These are elegant assays to test for antibody responses to defined epitopes, but require access to in vitro cultured parasites and monoclonal antibodies (mAb) making them difficult to include in most field studies. Additionally, the epitope recognized by the capture mAb is blocked and not accessible to the test antibodies. An alternative is to generate properly folded recombinant protein and test directly for immunoreactivity; however *Pfs*48/45 and *Pfs*230 both contain multiple 6-cysteine (6-cys) domain motifs which complicates the expression of correctly-folded recombinant proteins in heterologous systems [11–13]. The inclusion of a fusion partner such as maltose binding protein (MBP) [14] or *P. falciparum* glutamate rich protein (GLURP.R0) [11, 15] facilitates folding and expression of the 6-cys component. However, both GLURP.R0 and MBP are themselves immunogenic, requiring the antibody responses generated in an ELISA against the fusion partner to be subtracted from the overall response to the fusion protein [16]. *Pfs*230 has also been expressed using plant or cell-free wheat germ agglutinin expression systems, but both these methods require specialized techniques that are difficult to scale up [17, 18]. As an economical alternative to these methods, recombinant proteins that were specific for *Pfs*48/45 or *Pfs*230 were designed and produced using the *Lactococcus lactis* (*L. lactis*) expression system. This expression system has recently been used to produce properly folded chimeric GMZ2.48/45, which includes *Pfs*48/45 amino acid residues (aa 291–428), that induces transmission blocking antibodies in rats and is being developed as a vaccine candidate [11, 13]. A Tobacco Etch Virus (TEV) protease site was introduced between GMZ2 and *Pfs*48/45-6C components of the chimeric GMZ2.48/45 to allow the efficient removal of the fusion partner. The *L. lactis* expression system was also used to produce the N-terminal region (amino acid residues 443–590) of the processed form of *Pfs*230 that is retained on the gamete surface after erythrocyte emergence [17]. Previous work demonstrated that when this region of *Pfs*230, called C0, was produced in the wheat germ cell-free system the C0 recombinant protein induced antisera in mice that reduced the infectivity of *P. falciparum* to *Anopheles stephensi* mosquitoes in the presence of complement [17].

Both *L. lactis*-produced antigens were then used to determine immunoreactivity in 95 malaria patients in coastal Ghana. The results demonstrate that 74.7 and 72.8% of the individuals were seropositive for *Pfs*48/45-6C and *Pfs*230-C0_LI_, respectively, and there was a significant age-dependent increase in titer. This work demonstrates the natural immunogenicity of these important transmission-blocking targets, which suggests they could have the potential to boost a *Pfs*230 or *Pfs*48/45 vaccine response. The *L. lactis* antigens can also be used as tools for further analysis of the natural immune response against gametocytes.

## Methods

### Ethical statement

Archived sera used in this study were from a previous cross sectional study approved by the Institutional Review Board of the University of Cape Coast (UCCIRB/28/09/3.1.1) in addition to the Ethical Review Board of the Ministry of Health, Ghana MOH (GHS-ERC 17/01/12) and carried out in the Central Region of Ghana between 2011 and 2012. All participants and parents of minors were educated about the study and gave written consent prior to sample collection. All patient information is treated as confidential.

### Study site and sample acquisition

**Fig. 1:**
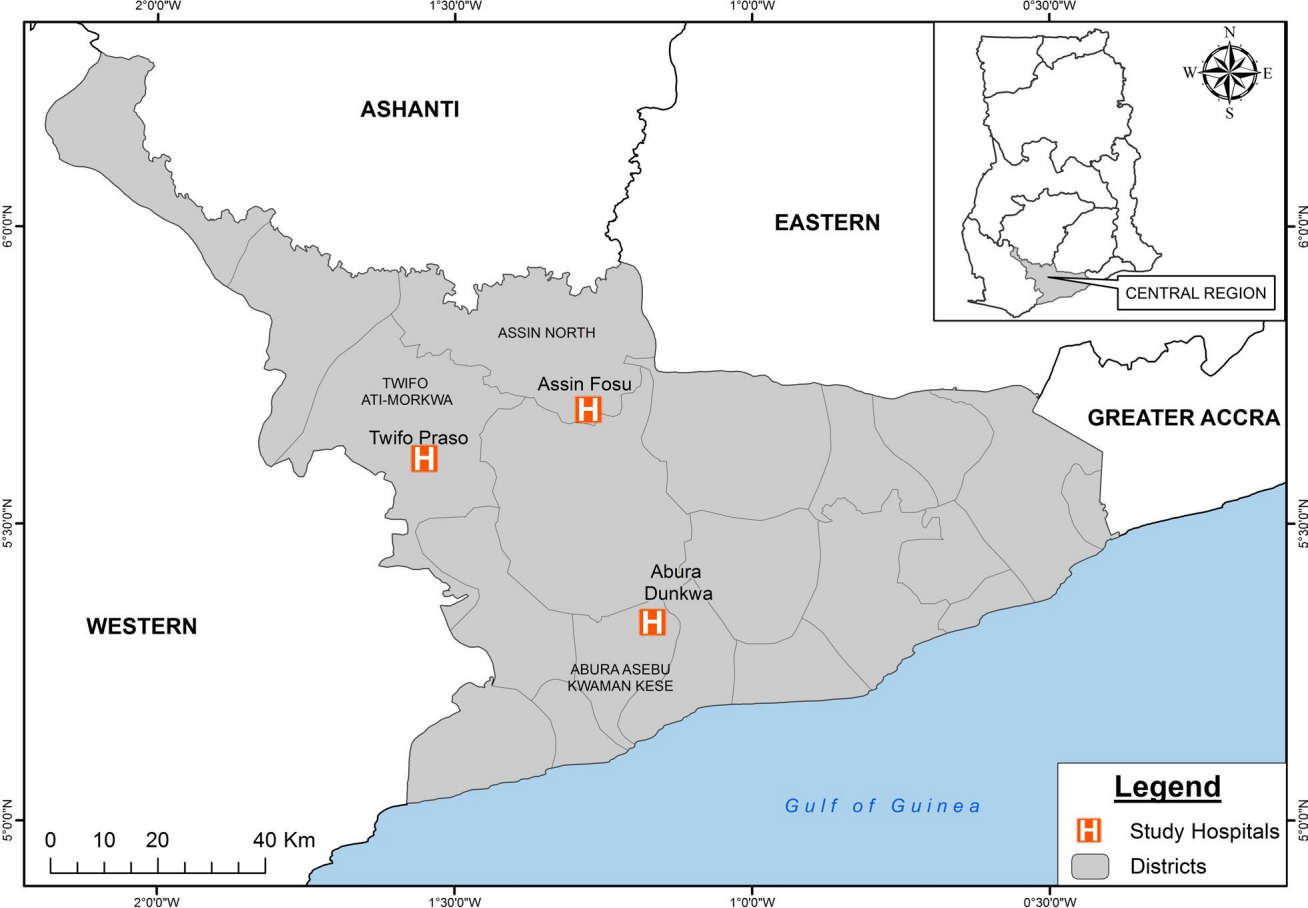
Map of the Central Region of Ghana showing the study districts

Samples for this study were collected from patients reporting to three district hospitals, one each in Abura Dunkwa, Twifo Praso and Assin Fosu, which are district capitals for the Abura-Asebu-Kwamankese, Twifo-Ati Morkwa and Assin North districts of the Central Region of Ghana (Fig. 1) from January to December 2012. These districts have been shown to harbor parasites with varying drug resistance susceptibilities [19] and a parasite prevalence ranging from 11.4 to 15.4% [20].

This study utilized paired archived filter paper blood blots and serum samples from 95 volunteers who had been confirmed as *P. falciparum* positive at the three district hospitals in a larger study conducted in 2012 and previously reported by Asare et al. [19].

### Plasmid constructs and protein production

#### *Pfs*230-C0_Ll_

**Fig. 2:**
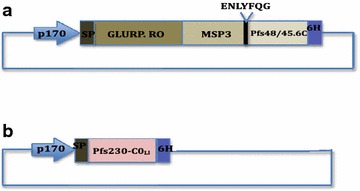
Schematic diagram of *Lactococcus lactis* based plasmids for expression of GMZ2.tev.6C (**a**) and *Pfs*230-C0_LI_ (**b**) proteins. Both plasmids contain the pH inducible P170 promoter and DNA sequences that attaches a hexahistidine tag to the C-terminus of the expressed proteins. The coding region for the TEV protease recognition sequence used was ENLYFQG

The nucleotides encoding *Pfs*230-C0_Ll_ (amino acid residues 443–590) were amplified from r230/MBP.C [21] using primers 230F443 and 230R590 (Additional file 1: Table S1) and Iproof™ High-Fidelity polymerase (Bio-Rad Laboratory, USA). The PCR amplicon was double digested with *Bgl*II and *BamH*I and ligated into *Bgl*II-linearized pLEA2 plasmid, a *L. lactis* expression vector which has the P170 pH sensitive promoter, the SP310mut2 secretion signal peptide [22] and also appends a C-terminal hexa-histidine tag to the expressed protein (Fig. 2). The ligated product was cloned in *E. coli* (*strain TOP10*) and the inserts were verified by sequencing (Macrogen Company, Holland). pLEA2 plasmid containing the *Pfs*230-C0_Ll_ coding sequence was transformed into *L. lactis* MG1363 strain by electroporation as previously described [23]. After overnight growth in a bio fermenter [11, 15], the *Pfs*230-C0_LI_ containing media was harvested by centrifugation at 9000×*g* for 15 min. The supernatant was filtered and diluted tenfold with equilibration buffer (50 mM Tris HCl, 200 mM NaCl, 25 mM imidazole, pH 8.0) and the *Pfs*230-C0_Ll_ protein was affinity-purified on a 5 ml HisTrap Crude FF column on the AKTA_XPRESS_ FPLC system. The column was washed with equilibration buffer to remove unbound proteins and then bound proteins were eluted with a 0–400 mM imidazole gradient in elution buffer (50 mM Tris HCl, 200 mM NaCl, 400 mM imidazole, pH 8.0) at a flow rate of 0.8 ml/min.

*Lactococcus lactis* expression plasmid, pGMZ2.tev.6C (Fig. 2), containing DNA encoding the Glutamine Rich Protein (GLURP.R0, nt 79–1500) and the Merozoite Surface Protein 3 (MSP3, nt 462–747) domain cassette and the C terminal region of *Pfs*48/45 (6C, nt 871–1284), with a TEV protease recognition site (ENLYFQG) at the MSP3 and 6C junction was constructed analogous to pGMZ2.6C [13]. However, the synthetic 285 bp MSP3 fragment was amplified with the TevF1 and TevR1 primers (Additional file 1: Table S1) using HotStar HiFidelity polymerase (Qiagen), to incorporate ENLYFQG at the 3′ end of the fragment prior to insertion into pSS2. The final plasmid, pGMZ2.tev.6C, containing the TEV protease site was transformed into *L. lactis* MG1363 [23] and fermented as previously described [15]. Culture medium containing secreted GMZ2.tev.6C protein was harvested, then concentrated tenfold and buffer exchanged into phosphate-buffered saline, pH 7.2 (PBS) containing 10 mM imidazole, using the QuixStand system (GE Healthcare, Sweden) followed by affinity purification using an HisTrap HP column (GE Healthcare) as previously described [11]. Recombinant TEV protease was produced and purified from expression vector, pRK793 as detailed by the manufacturer [24]. The purified GMZ2.tev.6C protein was digested overnight at 4 °C with a 1:10 concentration of TEV protease. The TEV-digested protein was diluted tenfold with 50 mM Tris, pH 8.0 and ion exchange chromatography on a 5 ml HiTrap Capto Q column (GE Healthcare) was performed for the separation of GMZ2, 6C and the TEV protease.

### Protein characterization

Crude or purified protein fractions were subjected to SDS-PAGE on 4–12% BisTris gel at 150 V for 1 h and stained with Coomassie blue or transferred to a hybond-ECL nitrocellulose membrane at 35 V for 1 h for subsequent immunoblotting. After transfer, the membrane was blocked with 5% skimmed milk in 1× PBS with 0.0 5% Tween 20 for 2 h before adding the indicated antibody. Horseradish peroxidase (HRP)-conjugated anti-His(C-term) antibodies (Thermo Scientific) were used to detect the presence of purified GMZ2.tev.6C and its digested products. The absence of any GLURP.R0 contaminant in the final purified cleaved 6C protein was determined by probing the immunoblot with polyclonal rabbit anti-GLURP.R0 antibodies and HRP conjugated anti-His(C-term) antibodies (Thermo Scientific). Polyclonal antibodies against *Escherichia coli* expressed *Pfs*230C followed with HRP-conjugated anti-mouse IgG were used to detect *Pfs*230-C0_Ll_. Pierce ECL Western Blotting Substrate (Thermo Scientific) or 3,3′-diaminobenzidine tetrahydrochloride (D5905, Sigma Life Sciences) used as a substrate for visualization. Folding of *Pfs*48/45-6C antigen was determined as previously described [11].

### *Pfs*48/45-6C and *Pfs*230-C0_Ll_ ELISA

NUNC Maxisorp 96-well ELISA plates were coated with 100 µl of 1 µg/ml of affinity purified *Pfs*230-C0_Ll_ or *Pfs*48/45-6C in carbonate buffer (0.05 M carbonate/bicarbonate buffer, pH 9.2) overnight at 4 °C. Plates were blocked with 150 µl of blocking buffer (3% skimmed milk in 1× PBS supplemented with 0.0 5% Tween 20 (PBST)) for 2 h. Following this, plates were washed three times with PBST and then incubated for 2 h at room temperature with 100 µl of test serum from malaria infected individuals (1/200 dilution in PBST) or a pool of negative control serum from donors living in non-endemic countries. The wells were then washed three times and 100 µl of polyclonal rabbit anti-human IgG-HRP (1/3000 dilution in 1× PBS with 0.0 5% Tween 20) was added to each well and incubated for 2 h. Plates were washed four times and then 100 µl of tetramethylbenzidine substrate solution was added per well and incubated for 20 min. Reactions were stopped using 100 µl per well of 0.2 M sulphuric acid and optical densities were measured at 450 nm using the ELx808 Absorbance Reader (BioTek). Optical densities (OD) of the serum samples obtained from each ELISA plate were transformed into IgG concentrations (ng/μl) based on the regression curve obtained from titrating purified human polyclonal IgG (PB055, The Binding Site) on the corresponding plate.

### Genomic DNA extraction, PCR and sequencing

Chelex in PBS was used to extract DNA from dried filter paper blood blots from 95 donors whose sera were used for ELISA [25]. Briefly, two 3 mm circles were cut from each blood blot using a 3 mm^2^ punches and placed in a 1.5 ml microcentrifuge tube. 1 ml of 0.02% saponin was added to each tube and agitated overnight (~17 h) at room temperature. The saponin solution was decanted and the blots washed with 1× PBS solution. 150 µl of a 6% Chelex/PBS solution was added to the washed blots, which were then incubated at 95 °C for 10 min in a water bathe. The extracted DNA served as a template for PCR amplification of fragments of both the *Pfs*48/45 and the *Pfs*230 genes, that encompass the regions of the genes used to produce the antigens used in this study. PCR amplicons were purified using the DNA clean and concentrator kits (Zymo) and subsequently sent together with the forward and reverse primers to MWGBiotech for bidirectional sequencing.

### Data analysis

Data was analysed using Microsoft Excel 2013 and GraphPad Prism v7 (GraphPad Software Inc.). Kruskal–Wallis test of variance and Duns test were used to determine significant difference among the mean values of *Pfs*48/45, *Pfs*230 antibodies and parasite density within the stratified study population. Shapiro–Wilk normality test was also used to test for normality in the distribution of log transformed antibody concentrations. ADAMSEL (Ed Remarque, BPRC) was used to analyze the ELISA data and convert the optical densities (OD) into concentration. BioEdit v7.2.6 (Tom Hall) and MAFFT online sequence alignment (Kazutaka Katoh) programs were used to assess the polymorphisms in the genes. Effects of mutations on protein sequence were also determined by EXPASY online translate tool [26].

## Results

### Recombinant *Pfs*48/45-6C and *Pfs*230-C0_Ll_ expression

**Fig. 3:**
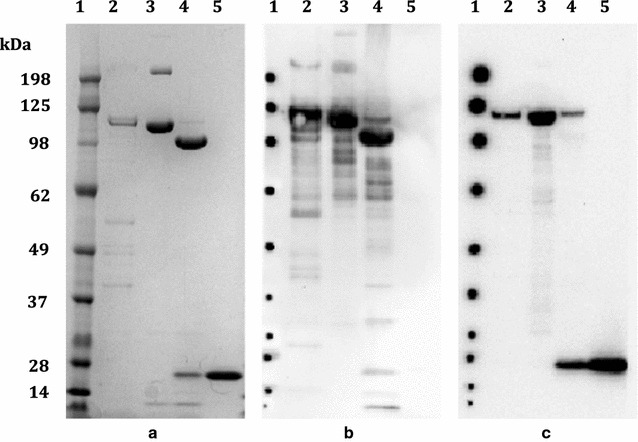
Expression and purification of *Pfs*48/45-6C by *L. lactis.* Culture supernatant (*lane 2*), Ni–NTA purified GMZ2.tev.6C protein (*lane 3*), TEV digested GMZ2.tev.6C (*lane 4*) and purified *Pfs*48/45.6C (*lane 5*) were subjected to SDS-PAGE and subsequently to Coomassie blue staining (**a**) or Western blotting using anti-GLURP.R0 (**b**) or anti-His(C-term) (**c**)

**Fig. 4:**
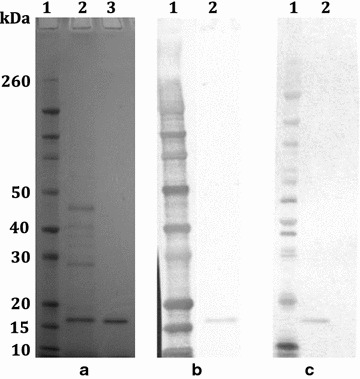
Expression of hexahistidine tagged *Pfs*230-C0_Ll_ by *L. lactis.* Culture supernatant (Gel A, *lane 2*), and purified antigens (*lane 3* of gel A and *lane 2* of gel B and C) were subjected to SDS-PAGE and subsequently stained with Coomassie blue (**a**) or western blotting with anti-His(C-term) (**b**) or anti-*Pfs*230C antibodies (**c**) as probe. Novex^®^ Sharp Prestained Protein Standard was used in *lane 1* of gel A, B and C

The nucleotides encoding the C terminal fragment of *Pfs*48/45 (bp 871–1284) containing epitope I, *Pfs48/45*-6C, and *Pfs*230 region C0 (bp 1329–1770), *Pfs*230-C0_Ll_, were successfully cloned using primers listed in Additional file 1: Table S1 into *L. lactis* expression plasmids pSS2 and pLEA2 to form the pGMZ2.tev.6C and p*Pfs*230-C0_Ll_ constructs, respectively (Fig. 2). Both recombinant proteins were successfully expressed by *L. lactis* (Figs. 3, 4) and purified using nickel-nitrilotriacetic acid (NTA) column. As expected the chimeric GMZ2/*Pfs*48/45-6C recombinant protein (GMZ2.tev.6C, yield 25 mg/l from overnight fermentation) strongly reacted with anti-GLURP.R0 (Fig. 3b, lanes 2 and 3) and HRP-conjugated anti-His(C-term) antibodies (Thermo Scientific) (Fig. 3c, lanes 2 and 3) prior to TEV protease treatment. TEV digestion generated two fragments of the expected sizes as well as a small amount of residual undigested full-length protein (Fig. 3a, b, lane 4). This residual undigested full-length protein still retains the C-terminal Histidine (His) tag and is detected with anti-His(C-term) antibodies (Fig. 3c, lane 4). The released *Pfs*48/45.6C fragment was subsequently purified over the CaptoQ column (GE Healthcare Life Sciences) (Fig. 3, lane 5) with a yield of 5 mg/l. The purified *Pfs*48/45.6C antigen did not react with polyclonal GLURP.R0 antibodies (Fig. 3b, lane 5) but reacted with anti-His(C-term) antibodies (Fig. 3c, lane 5), suggesting that there was no GLURP.R0 contamination in the final product.

*Lactococcus lactis* expressed *Pfs*230-C0_Ll_ was successfully purified in a single step using a HisTrap Crude FF column (Fig. 4) yielding 15.4 mg/l of pure protein from culture medium harvested after overnight growth without fermentation. Affinity purified *Pfs*230-C0_LI_ was recognized by antibodies against a C-terminal polyhistidine tag (Fig. 4b, lane 2) and mouse polyclonal antibodies generated against *E. coli* expressed *Pfs*230C recombinant protein (Fig. 4c, lane 2) [7].

Both recombinant proteins were used to evaluate the immune response against these sexual stage antigens. The study population included 95 symptomatic malaria patients, 20 (21.1%) of the samples were from volunteers resident in Abura Dunkwa, 44 (46.3%) were from Assin Fosu and 31 (32.6%) from Twifo Praso. The geometric mean age of the study population was 7.045 (CI 5.56–8.92) years and geometric mean *P. falciparum* parasite density of 7464 (CI 5227–10,659) parasites**/**μl. As is typical in many field studies, where the main interest is asexual parasite density, gametocyte data was not recorded in any of the thick smears prepared from the study participants. 65% of the volunteers were female (Table 1). Although there was no significant difference between parasite densities across the study population, there was a general trend of decreased parasite load in the older population compared to children 5 years and below (P = 0.107) (Table 1). A similar trend was seen with haemoglobin concentrations as might be expected since PD can influence HB. The geometric mean of the hemoglobin concentrations within the entire study population was 9.54 (CI 9.16–9.93) g/dl (Table 1), while the geometric mean level in children 5 years and below was lower at 8.93 (CI 8.27–9.65) g/dl but not significantly different (P = 0.072) from the older children or the adults (Table 1).

**Table 1:** Clinical features of participants at presentation

	HB (g/dl)				Age (years)				PD (/µl)			
	Total	0–5	6–17	≥18	Total	0–5	6–17	≥18	Total	0–5	6–17	≥18
Count	90	37	23	30	95	39	23	33	90	36	23	31
Minimum	4.4	4.4	7	7.3	0.33	0.33	6	18	40	40	680	240
Maximum	13.6	12	13.6	11.6	35	5	17	35	184,600	184,600	109,080	84,000
Geometric mean (GM)	9.54	8.93	10.04	9.95	7.045	2.162	9.59	22.94	7464	9313	10,271	4555
Lower 95% CI of GM	9.16	8.27	9.28	9.54	5.56	1.71	8.10	21.5	5227	4957	5478	2530
Upper 95% CI of GM	9.93	9.65	10.86	10.37	8.92	2.72	11.35	24.49	10,659	17,498	19,259	8202

### Seroprevalence of antibodies against recombinant *Pfs*230 and *Pfs*48/45 antigens

**Fig. 5:**
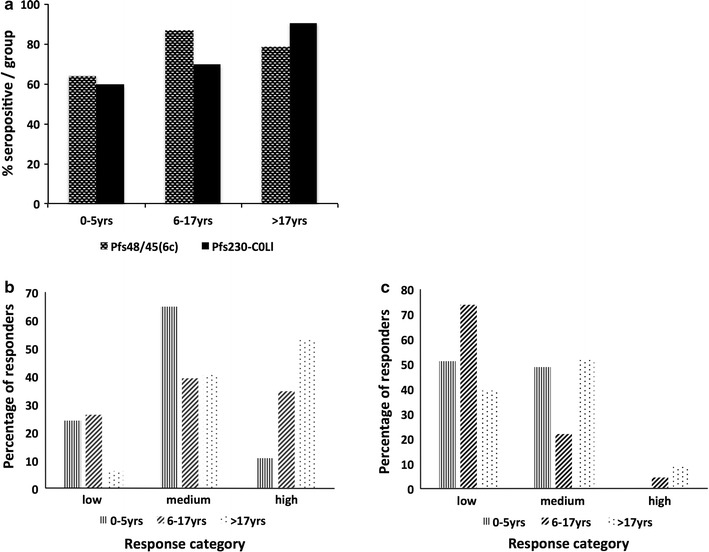
Seroprevalence of anti-Pfs48/45.6C and Pfs230-C0_Ll_ antibodies. **a** The proportion of young children (0–5 years), older children (6–17 years) and adults (above 17 years) who were seropositive for *Pfs*48/45 and *Pfs*230 using *L. lactis* expressed *Pfs*48/45-6C and Pfs230-C0_Ll_ antigens. Antibody responses to P*fs*48/45-6C (**b**) and *Pfs*230-C0_LI_ (**c**) were categorized as low (≤twofold higher than the negative control cutoff, medium (between two and fourfold higher than the negative control cutoff) or high (≥fourfold higher than the negative control cutoff). The percentage of young children, older children and adults that were seropositive with low, medium and high antibody levels against *Pfs*48/45-6C (**b**) or *Pfs*230-C0_Ll_ (**c**) were plotted. The exact number of patients sampled in each age group is listed in Additional file 2: Table S2

**Fig. 6:**
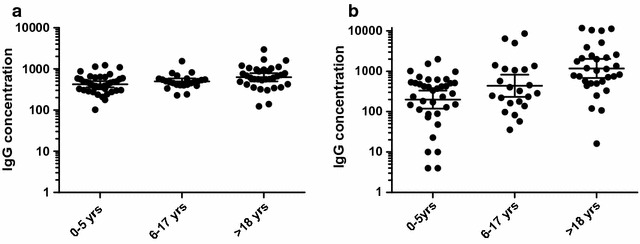
Antibodies titers against *L. lactis* expressed Pfs48/45-6C (**a**) and *Pfs*230C0_Ll_ (**b**) antigens, respectively in the total study population. An ELISA was used to test recombinant antigens for reactivity with serum from malaria infected children and adults living in the Central Region of Ghana. All serum samples (1:200 dilution) were tested in duplicate and repeated at least twice. Optical densities (OD) for the serum samples were obtained from each ELISA plate and transformed into IgG concentrations (ng/μl) based on the regression curve obtained from titrating purified human polyclonal IgG (PB055, The Binding Site) on the corresponding plate. The graph shows the geometric mean of the antibody concentrations plus or minus the 95% confidence interval. The exact number of patients sampled in each age group is listed in Additional file 2: Table S2

This study set out to utilize newly produced sexual stage antigens to determine whether the immune response in malaria patients in the Central Region of Ghana varied with age, PD or HB levels, which have been associated with increased gametocyte carriage [27, 28]. The percentages of malaria-infected individuals who had antibody responses against *Pfs*230-C0_Ll_ or *Pfs*48/45-6C that were (two standard deviations) higher than those observed for the pooled negative control serum (159 ± 55.4 and 312 ± 46.6 ng/ml), respectively, were determined in an indirect ELISA. It was observed that 72.8% of the malaria-infected study population was seropositive for *Pfs*230-C0_Ll_ while 74.7% were seropositive for *Pfs*48/45-6C antigen. The seroprevalence was similar in different age groups (P = 0.10) (Fig. 5a), however adults (above 17 years) had significantly higher antibody [29] levels against both *Pfs*48/45-6C and *Pfs*230-C0_Ll_ than the young children (0–5 years) with P values of 0.0037 and less than 0.0001, respectively (Fig. 6a; Additional file 2: Table S2). Consistent with an age dependent response, only individuals older than 5 years of age had anti-*Pfs*48/45-6C titers that were fourfold or more above the pooled negative serum (high responders) (Fig. 5c). A broader range of titers was obtained against *Pfs*230-C0_Ll_, with all ages represented in the high responder category (Fig. 5b). Older children and adults were still the majority of the high *Pfs*230-C0_Ll_ responders, while younger children were more likely to be low or medium responders and older children were equally distributed between all three categories.

### Gene polymorphisms

To evaluate whether antigen polymorphisms in the local parasite populations could affect immunoreactivity against the recombinant proteins that corresponded to lab strain 3D7 parasites, the corresponding genes were amplified from DNA obtained from subject blood samples. Polymorphisms in the gene fragments encoding *Pfs*230-C0_Ll_ and *Pfs*48/45-6C were assessed using primers listed in Additional file 1: Table S1. Polymorphisms observed are summarized in Table 2. The main polymorphism identified in the *Pfs*230-C0 region was a deletion of nucleotides 1559–1567 which occurred in 4 out of the 13 successfully sequenced samples (Table 2; Additional file 4: Figure S1). This polymorphism deletes one of the two tandem ‘YGE’ sequences, but does not change the reading frame. In *Pfs*48/45, the mutation identified was a T940A, which has the downstream effect of changing leucine to isoleucine in the protein and occurred in 12 out of 19 sequenced DNA samples (Table 2; Additional file 4: Figure S1). The antibodies from subjects infected with parasites with these mutations still recognized *Pfs*48/45-6C and *Pfs*230-C0_Ll_ (Additional file 3: Table S3), suggesting the changes did not have a major impact on the immune response of these subjects or that the antibodies were elicited by a previous infection.

**Table 2:** Mutations in genes encoding *Pfs*230-C0_Ll_ and *Pfs*48/45-6C

Antigen	Mutation	Translation	Frequency
Pfs48/45-6C	T940A	L → I	12/19
Pfs230-C0Ll	1559–1567	‘YGE’ deletion	4/13

Frequency is defined as the number of samples with mutation/total successfully sequenced samples

## Discussion

This study demonstrates the successful expression of *Pfs*230- and *Pfs*48/45-specific antigens, *Pfs*230-C0_Ll_ and *Pfs*48/45-6C, respectively. The parental chimeric *Pfs*48/45-6C has been shown to induce antibodies that inhibit malaria transmission in a SMFA [11] and TEV cleavage was not found to alter that structure, while *Pfs*230-C0_Ll_ reacts with antibodies produced against recombinant *Pfs*230 region C, which have transmission-blocking activity [30]. Together the two antigens provide new, economical tools to monitor immunoreactivity in human blood samples. *L. lactis* is a current good manufacturing practice (cGMP), scalable expression system that allowed the purification of *Pfs*230-C0_Ll_ antigen from culture media in one step to yield 1.54 mg of pure protein from a 100 ml of an overnight benchtop culture. It is likely that much higher yields could be obtained if the construct is fermented in a 1l biofermentor. Secretion of the recombinant protein into the culture media simplifies purification by bypassing the need to lyse the bacteria, making the process more consistent as well as less laborious. The *Pfs*48/45-6C domain was produced as a fusion with GLURP.R0/MSP3, purified from the culture media using Nickel-resin at 25 mg/l and then efficiently released from GLURP.R0/MSP3 using TEV protease and repurified by ion exchange. Initially a mAb affinity purification step was included after the ion exchange chromatography, but our desire to produce a scalable antigen and our experience from the purification of GLURP.R0.6C [11] lead us to do away with the unscaleable mAb purification step, which did not significantly enhance the purity.

A recent study conducted in the Central Region of Ghana identified a high proportion of children who harbored submicroscopic gametocytes [31], but did not investigate how gametocyte carriage influences the acquisition of antibodies against these life stages of the parasite. This study thus set out to utilize newly produced sexual stage antigens to determine the baseline responses individuals in the Central Region of Ghana generate against the selected gametocyte antigens.

Using both purified antigens to evaluate natural antibody responses in malaria patients from the Central Region of Ghana, the seroprevalence of antibodies against *Pfs*230-C0_Ll_ and *Pfs*48/45-6C was 72.8 and 74.7% respectively. The similar seroprevalence rates against these two sexual stage antigens is consistent with the similar gametocyte-specific expression patterns of *Pfs*230 and *Pfs*48/45 and suggests that the immune response may be associated with gametocyte exposure, as was previously reported in a longitudinal study in Tanzania using a two-site ELISA with purified gametocyte extract as antigen [10]. A significant positive association between the concentrations of antibodies recognizing *Pfs*230-C0_Ll_ and *Pfs*48/45-6C in individuals (r = 0.378, P = 0.0002) is further support for a coordinated immune response against the two antigens, which would be expected if gametocyte exposure stimulated immunoreactivity. For both antigens, antibody titers increased significantly with age, which is suggestive of the development of immunological memory that could be boosted by exposure to a vaccine containing similar regions of Pfs230 or Pfs48/45 or natural exposure to the sexual stages of the malaria parasite. Age-dependence of *Pfs*230-C0_Ll_ and *Pfs*48/45-6C antibody responses was also reported in the longitudinal study in Tanzania mentioned above and in Irian Jayian transmigrants [32], but has not been observed in a number of other studies evaluating *Pfs*230 and *Pfs*48/45 using a two site ELISA [8, 9]. A recent cross-sectional study of healthy school children in the Eastern region of Ghana, reported low Pfs230 and Pfs48/45 antibody seroprevalence, 20.7 and 15.2%, respectively and no age dependence using recombinant Pfs48/45 region 10C (aa 172–428) and Pfs230 region CMB (aa 444–730) [33]. The difference between this cross sectional study and our evaluation of malaria patients, could be due in part to a relatively short-lived antibody responses to *Pfs*230 and *Pfs*48/45, which have been reported previously [8, 34]. The evaluation of malaria patients that are likely to have a high prevalence of submicroscopic levels of gametocytes could contribute to the high seroprevalence observed. To address this directly, longitudinal studies are needed that monitor gametocyte exposure and the responses of the same children before and after an infection. Understanding the persistence of antibody responses in the presence and absence of parasite exposure is critical to vaccine design and administration. In the future it will also be important to directly determine whether the specific human antibodies that recognize these antigens have transmission-blocking activity.

Antigenic polymorphisms are also an important consideration when evaluating field samples. Although, more limited than some asexual stage surface antigens, sexual stage antigens have been found to be polymorphic [35]. Genomic DNA sequence from parasites isolated from the blood of 19 subjects revealed a T940A mutation in the 6C region (bp 859–1284) of *Pfs*48/45 (Table 2; Additional file 4: Figure S1) in 12 individuals. This mutation changes leucine (aa 314) to isoleucine, another hydrophobic amino acid and is also a predominant mutation in other field samples [36]. No other *Pfs*48/45 mutation was found in the region of the isolates that were sequenced in this study. The mutation identified in the C0 region (bp 1329–1764) of Pfs230 was a deletion of 9 nucleotides 1559–1567 in four samples (Table 2; Additional file 4: Figure S1). This mutation deletes one of the duplicated YGEs (aa 521–526) in the 3D7 reference parasite sequence. Deletions or additions in regions of tandem amino acid repeats are not uncommon in the *Pfs*230 gene sequence [17]. These small changes were not found to have a major impact on the immune response observed in this study against *Pfs*230-C0_Ll_ and *Pfs*48/45-6C (Additional file 3: Table S3).

## Conclusion

*Plasmodium falciparum* sexual stage antigens, *Pfs*230-C0_Ll_ and *Pfs*48/45-6C have been produced using in a scalable, *L. lactis* expression system that can be used as tools for monitoring natural immune responses. In addition to high antibody prevalence in malaria patients in the Central region of Ghana, antibody titers increased significantly with age, which is consistent with the development of immunological memory against these important transmission-blocking candidates.

## Additional files

**Additional file 1: Table S1.** Primes used for cloning and sequencing.

**Additional file 2: Table S2.** Features of samples used for ELISA.

**Additional file 3: Table S3.** Seroreactivity of sera from individuals infected with mutant parasites.

**Additional file 4: Figure S1.** Antigenic polymorphisms.

## Abbreviations

Aa: amino acids
ACT: artemisinin-based combination therapy
bp: base pairs
C-term: carboxy-terminus
DNA: deoxyribonucleic acid
HRP: horseradish peroxidase
nt: nucleotide
PD: parasite density
PCR: polymerase chain reaction
*Pfs*48/45: *Plasmodium falciparum* sexual-stage antigen (PF3D7_1346700)
*Pfs*230: *Plasmodium falciparum* sexual-stage antigen (PF3D7_0209000)
TEV: Tobacco Etch Virus
