# Supplementary material for: Antibody responses to two new Lactococcus lactis-produced recombinant Pfs48/45 and Pfs230 proteins increase with age in malaria patients living in the Central Region of Ghana
Source: Malar J. 2017 Aug 1;16:306. doi: 10.1186/s12936-017-1955-0 (PMC5540549; doi:10.1186/s12936-017-1955-0)
Supplement: Supplementary file 1 — Additional file 1: Table S1. Primes used for cloning and sequencing. [file 12936_2017_1955_MOESM1_ESM.docx]

**SUPPLEMENTARY DATA**

Supplementary table1: Primes used for cloning and sequencing

| **ANTIGEN** | **PRIMER** | **SEQUENCE** |
| --- | --- | --- |
| **Cloning** |  |  |
| **6C** | TEVF1 | 5’-AGACTTCTACTATTTTGTTGTCTAGTAGGATCC-3’ |
|  | TEVR1 | 5’-GTACAGATCTGCCCTGAAAATACAGGTTCTCTTCAAGTTCTTCTTCTTC TGTTTCTTCTGC-3’ |
| **Pfs230** | 230F443 | 5’-TAATGGATCCGAATATGTAGATGAAAAAGAA-3’ |
|  | 230R590 | 5’-CCATAGATCTTTCTTTATTTGTATTATTAGA-3’ |
|  |  |  |
| **Sequencing** |  |  |
| **6C** | 4845ORI | 5’-AGCCAAAAATCCATAATAA-3’ |
|  | 4845SFI | 5’-TCGGAATTAGGTTTAATTGAATATG-3’ |
| ***Pfs*230-C0_LI_** | 230F443 | 5’-TAATGGATCCGAATATGTAGATGAAAAAGAA-3’ |
|  | 230R590 | 5’-CCATAGATCTTTCTTTATTTGTATTATTAGA-3’ |
