# Supplementary material for: Antibody responses to two new Lactococcus lactis-produced recombinant Pfs48/45 and Pfs230 proteins increase with age in malaria patients living in the Central Region of Ghana
Source: Malar J. 2017 Aug 1;16:306. doi: 10.1186/s12936-017-1955-0 (PMC5540549; doi:10.1186/s12936-017-1955-0)
Supplement: Supplementary file 2 — Additional file 2: Table S2. Features of samples used for ELISA. [file 12936_2017_1955_MOESM2_ESM.docx]

Supplementary Table 2: Features of samples used for ELISA

|  |  | PFS48/45 | | | PFS230 | | |
| --- | --- | --- | --- | --- | --- | --- | --- |
|  |  | 0-5 (39) | 6-17 (23) | >17 (33) | 0-5 (37) | 6-17 (23) | >17 (32) |
| COUNT |  | 38 | 23 | 33 | 31 | 23 | 31 |
| Minimum titre | | 166 | 354.5 | 306.1 | 29.1 | 96.17 | 270.6 |
| Maximum titre | | 2520 | 3429 | 5879 | 2371 | 23135 | 48855 |
| Geometric mean (GM) titre | | 766.5 | 932.8 | 1309 | 520 | 776 | 2590 |
| Lower 95% CI of GM titre | | 628.1 | 722.1 | 1029 | 364.5 | 434.5 | 1608 |
| Upper 95% CI of GM titre | | 935.5 | 1205 | 1664 | 741.9 | 1386 | 4172 |

CI, confidence interval. Samples are stratified by age into three cohorts 0-5, 6-17 and greater than 17 years with the total number in each age group stated in parenthesis below the age group. The values in the table represent antibody concentrations in ng/ml.
