# Supplementary material for: Antibody responses to two new Lactococcus lactis-produced recombinant Pfs48/45 and Pfs230 proteins increase with age in malaria patients living in the Central Region of Ghana
Source: Malar J. 2017 Aug 1;16:306. doi: 10.1186/s12936-017-1955-0 (PMC5540549; doi:10.1186/s12936-017-1955-0)
Supplement: Supplementary file 3 — Additional file 3: Table S3. Seroreactivity of sera from individuals infected with mutant parasites. [file 12936_2017_1955_MOESM3_ESM.docx]

**Supplementary Table 3: Seroreactivity of sera from individuals infected with mutant parasites**

| Mutation | Seroreactivity |
| --- | --- |
| Pfs230 YGE deletion |  |
| T164 | Positive |
| T181 | Negative |
| S078 | Negative |
| S129 | Negative |
| PfS4845 T-A mutation |  |
| A9308 | Positive |
| T120 | Positive |
| T118 | Negative |
| T186 | Positive |
| A9049 | Positive |
| T135 | Positive |
| S030 | Positive |
| S072 | Positive |
| S070 | Positive |
| S082 | Positive |
| S035 | Positive |
| S073 | Positive |
| S024 | Positive |
| S043 | Positive |
| T158 | Negative |
