# Supplementary material for: Antibody responses to two new Lactococcus lactis-produced recombinant Pfs48/45 and Pfs230 proteins increase with age in malaria patients living in the Central Region of Ghana
Source: Malar J. 2017 Aug 1;16:306. doi: 10.1186/s12936-017-1955-0 (PMC5540549; doi:10.1186/s12936-017-1955-0)
Supplement: Supplementary file 4 — Additional file 4: Figure S1. Antigenic polymorphisms. [file 12936_2017_1955_MOESM4_ESM.docx]

Supplementary Fig. 1 Antigenic polymorphisms

BioEdit preview of MAFFT aligned sequences of Pfs48/45 amplicons from *P. falciparum* parasites.

....|....| ....|....| ....|....| ....|....| ....|....|

910 920 930 940 950

**3D7**  TCTTCAAATG TTAGTTCTAA ACATACTTTT ACAGATAGTT TAGATATTTC

**Pf4845-1-P** TCTTCAAATG TTAGTTCTAA ACATACTTTT ACAGATAGTT TAGATATTTC

**Pf4845-16-** TCTTCAAATG TTAGTTCTAA ACATACTTTT ACAGATAGTT TAGATATTTC

**Pf4845-3-P** TCTTCAAATG TTAGTTCTAA ACATACTTTT ACAGATAGTA TAGATATTTC

**Pf4845-9-P** TCTTCAAATG TTAGTTCTAA ACATACTTTT ACAGATAGTA TAGATATTTC

**Pf4845-4-P** TCTTCAAATG TTAGTTCTAA ACATACTTTT ACAGATAGTA TAGATATTTC

**Pf4845-5-P** TCTTCAAATG TTAGTTCTAA ACATACTTTT ACAGATAGTA TAGATATTTC

**Pf4845-17-** TCTTCAAATG TTAGTTCTAA ACATACTTTT ACAGATAGTA TAGATATTTC

**Pf4845-7-P** TCTTCAAATG TTAGTTCTAA ACATACTTTT ACAGATAGTA TAGATATTTC

**Pf4845-11-** TCTTCAAATG TTAGTTCTAA ACATACTTTT ACAGATAGTT TAGATATTTC

**Pf4845-14-** TCTTCAAATG TTAGTTCTAA ACATACTTTT ACAGATAGTT TAGATATTTC

**Pf4845-2-P** TCTTCAAATG TTAGTTCTAA ACATACTTTT ACAGATAGTA TAGATATTTC

**Pf4845-15-** TCTTCAAATG TTAGTTCTAA ACATACTTTT ACAGATAGTA TAGATATTTC

**Pf4845-13-** TCTTCAAATG TTAGTTCTAA ACATACTTTT ACAGATAGTA TAGATATTTC

**Pf4845-20-** TCTTCAAATG TTAGTTCTAA ACATACTTTT ACAGATAGTT TAGATATTTC

**Pf4845-10-** TCTTCAAATG TTAGTTCTAA ACATACTTTT ACAGATAGTA TAGATATTTC

**Pf4845-12-** TCTTCAAATG TTAGTTCTAA ACATACTTTT ACAGATAGTA TAGATATTTC

**Pf4845-18-** TCTTCAAATG TTAGTTCTAA ACATACTTTT ACAGATAGTA TAGATATTTC

**Pf4845-19-** TCTTCAAATG ATAGTTCTAA ACATACTTTT ACAGATAGTT TAGATATTTC

**Pf4845-8-P** TCTTCAAATG TTAGTTCTAA ACATACTTTT ACAGATAGTT TAGATATTTC

BioEdit preview of MAFFT aligned sequences of Pfs230 amplicons from *P. falciparum* parasites

....|....| ....|....| ....|....| ....|....| ....|....|

1560 1570 1580 1590 1600

**3D7**  TGCACGAGAT GGTGAATATG GTGAATATGG TGAAGCTGTC GAAGATGGAG

**AD9157-230** TGCACGAGAT GGTGAATATG GTGAATATGG TGAAGCTGTC GAAGATGGAG

**AD9602-230** TGCACGAGAT GGTGAATATG GTGAATATGG TGAAGCTGTC GAAGATGGAG

**AD9308-230** TGCACGAGAT GGTGAATATG GTGAATATGG TGAAGCTGTC GAAGATGGAG

**S036-230.S** TGCACGAGAT GGTGAATATG GTGAATATGG TGAAGCTGTC GAAGATGGAG

**S077-230.S** TGCACGAGAT GGTGAATATG GTGAATATGG TGAAGCTGTC GAAGATGGAG

**S030-230.S** TGCACGAGAT GGTGAATATG GTGAATATGG TGAAGCTGTC GAAGATGGAG

**AD9535-230** TGCACGAGAT GGTGAATATG GTGAATATGG TGAAGCTGTC GAAGATGGAG

**S141-230.S** TGCACGAGAT GGTGAATATG GTGAATATGG TGAAGCTGTC GAAGATGGAG

**AD9482-230** TGCACGAGAT GGTGAATATG GTGAATATGG TGAAGCTGTC GAAGATGGAG

**T164-230.S** TGCACGAG-- -------ATG GTGAATATGG TGAAGCTGTC GAAGATGGAG

**T181-230.S** TGCACGAG-- -------ATG GTGAATATGG TGAAGCTGTC GAAGATGGAG

**S078-230.S** TGCACGAG-- -------ATG GTGAATATGG TGAAGCTGTC GAAGATGGAG

**S129-230.S** TGCACGAG-- -------ATG GTGAATATGG TGAAGCTGTC GAAGATGGAG
